# Supplementary material for: An optimized transformer model for efficient detection of thoracic diseases in chest X-rays with multi-scale feature fusion
Source: PLoS One. 2025 May 7;20(5):e0323239. doi: 10.1371/journal.pone.0323239 (PMC12058152; doi:10.1371/journal.pone.0323239)
Supplement: Attachment — Submitted filename: Response.docx [file pone.0323239.s002.docx]

Reviewer #1:

1. In the abstract, the authors should mention how much improvement the proposed model achieved compared to existing methods.

**Response:** Thanks for your comment. We have added the following description in the abstract.

Experimental results on the NIH Chest X-ray dataset demonstrate that CD-DETR achieves a precision of 88.3% and recall of 86.6%, outperforming other DETR variants by an average of 5% and CNN-based models like YOLOv7 by 6-8% in these metrics, showing its potential for practical application in medical imaging diagnostics.

2. At the end of the introduction section, the authors should mention the main contributions of the proposed model in points.

**Response:** We agree with your opinion. Clearly listing the main contributions at the end of the introduction will improve readability and highlight the significance of the research. We have added the following in the Introduction section.

The main contributions of this study are as follows:

- **Development of CD-DETR**: We propose an optimized Detection Transformer model, CD-DETR, tailored for CXR analysis, addressing the limitations of standard DETR in detecting small and subtle thoracic pathologies.
- **Multi-Scale Feature Fusion**: We introduce a novel fusion strategy combining Efficient Channel Attention (ECA-Net) and Spatial Attention Upsampling (SAU), enhancing the model’s ability to capture contextual information across scales.
- **CDIoU Loss Function**: We design a specialized Chest Diseases Intersection over Union (CDIoU) loss function to improve small object detection and mitigate class imbalance, accelerating training stability.
- **Practical Impact**: We demonstrate the model’s applicability in AI-assisted centralized image reading systems, improving diagnostic efficiency in resource-scarce regions, with superior performance validated on benchmark datasets.

3. In the related work, the authors need to cite all the text with a supporting article properly.

**Response:** We acknowledge that the "Related Works" section contains some statements lacking citations, and we have made modifications and additions to this.

4. The authors need to provide all the parameters used for model training in the form of a table.

**Response:** We have added the "4.2 Training Setup" chapter as follows.

4.2 Training Setup

The CD-DETR model was trained using the PyTorch framework on an NVIDIA A100 GPU with 40GB memory. In the experiment, we utilized a Resnet model[29] pre-trained on ImageNet as the backbone network for extracting multi-scale features. The training configuration is detailed in Table 1 below to ensure reproducibility.

**Table 1.** Training parameters for CD-DETR model.

| Parameter | Value |
| --- | --- |
| Backbone Network | ResNet-50 |
| Optimizer | AdamW |
| Learning Rate | 0.0002 |
| Weight decay | 0.0001 |
| Batch Size | 16 |
| Epochs | 72 |
| Number of Encoder Layers | 6 |
| Number of Decoder Layers | 6 |

5. The authors need to compare the results of the proposed model with the existing state-of-the-art model to validate its effectiveness.

**Response:** The paper compares CD-DETR with DETR variants (e.g., Deformable-DETR) and CNN-based models (e.g., Hybrid-YOLO) in Tables 2 and 3. However, the comparison could be expanded to include more recent state-of-the-art (SOTA) models beyond those listed, such as YOLOv8, to strengthen the validation.

6. The authors need to cite the recent deep learning-based predictors such as pACP-HybDeep, TargetCLP, DeepAIPs-Pred, and AIPs-DeepEnC-GA to provide an updated overview to the readers.

**Response:** We have carefully read the papers on the suggested predictors (pACP-HybDeep, TargetCLP, DeepAIPs-Pred, AIPs-DeepEnC-GA), and we discussed their technical characteristics in the "Limitations and Prospects " chapter and will utilize their technological advantages in future research.

7. How the authors evaluate the overfitting of the proposed model.

**Response:** We thank the reviewer for raising this important point. While we did not explicitly discuss overfitting evaluation in the original manuscript, we monitored it during training using standard practices such as train-validation split and performance comparison on held-out test sets (NIH and VinBigData datasets). Overfitting was mitigated through techniques like data augmentation and weight decay, but this was not detailed. We hanve revised Section 4 to include a subsection on overfitting evaluation, clarifying our approach and results as follows.

To assess overfitting, we split the NIH Chest X-ray dataset into 80% training, 10% validation, and 10% test sets, and the VinBigData sample similarly. Training and validation losses were monitored over 72 epochs, with convergence observed around epoch 60, indicating no significant divergence (validation loss stabilized at ~0.15 vs. training loss ~0.12). Techniques such as random flipping and rotation for data augmentation, combined with a weight decay of 0.0001 in the AdamW optimizer, further prevented overfitting. Test set performance (88.3% precision, 86.6% recall on NIH) closely aligned with validation results, confirming the model’s generalization ability.

8. What should be the future directions of the proposed study?

**Response:** Future work could include real-time deployment, broader dataset validation, and integration into clinical workflows. We have revised Section 5.2 to elaborate on these directions.

In summary, future directions for this study include:

- Real-Time Deployment: Optimize CD-DETR for edge devices using techniques like model pruning or quantization to enable real-time inference in low-resource settings.
- Global Dataset Validation: Expand evaluation to include CXR datasets from diverse regions (e.g., Africa, South America) to ensure robustness across patient populations and imaging conditions.
- Clinical Integration: Develop a clinical decision support system (CDSS) incorporating CD-DETR, with user-friendly interfaces and compatibility with hospital systems (e.g., PACS), to enhance adoption in centralized reading workflows.
- Advanced Feature Extraction: Explore novel attention mechanisms or background suppression techniques to further improve detection in complex CXR images with overlapping structures.

Reviewer #2:

1. Check for grammatical, spelling, and punctuation errors.

**Response:** Thank you for highlighting the importance of linguistic accuracy. We will conduct a thorough proofreading pass to correct these errors and ensure the text adheres to standard academic English conventions.

2. Use more significant keywords.

**Response:** We will revise the keyword list to better reflect the study’s scope and technical contributions.

3. Write the result clearly in the abstract.

**Response:** Thank you for this feedback. We revised the abstract to include quantitative results from the experiments as follows.

Experimental results on the NIH Chest X-ray dataset demonstrate that CD-DETR achieves a precision of 88.3% and recall of 86.6%, outperforming other DETR variants by an average of 5% and CNN-based models like YOLOv7 by 6-8% in these metrics, showcasing its potential for practical application in medical imaging diagnostics.

4. There are too many paragraphs in the introduction. Maximum 5-6 paragraphs are good.

**Response:** To improve readability and conciseness, we will consolidate related content (e.g., medical resource disparity and centralized reading model) into fewer paragraphs, targeting a maximum of 6 paragraphs while retaining all key information.

5. Write the novelty/contribution of this work clearly in the introduction section.

**Response:** We appreciate this suggestion, and we added a concise summary at the end of the introduction to clarify the significance.

The main contributions of this study are as follows:

- **Development of CD-DETR**: We propose an optimized Detection Transformer model, CD-DETR, tailored for CXR analysis, addressing the limitations of standard DETR in detecting small and subtle thoracic pathologies.
- **Multi-Scale Feature Fusion**: We introduce a novel fusion strategy combining Efficient Channel Attention (ECA-Net) and Spatial Attention Upsampling (SAU), enhancing the model’s ability to capture contextual information across scales.
- **CDIoU Loss Function**: We design a specialized Chest Diseases Intersection over Union (CDIoU) loss function to improve small object detection and mitigate class imbalance, accelerating training stability.
- **Practical Impact**: We demonstrate the model’s applicability in AI-assisted centralized image reading systems, improving diagnostic efficiency in resource-scarce regions, with superior performance validated on benchmark datasets.

6. Use a summary table for previous works in the related works.

**Response:** Thank you for this suggestion to improve the organization of Section 2. A summary table will provide a clearer, more concise overview of prior works compared to the current narrative format. We created a table summarizing key methods, their approaches, and limitations relevant to CXR recognition

Table 1: Summary of Previous Works in CXR Image Recognition.

| Method | Approach | Strengths | Limitations | Ref |
| --- | --- | --- | --- | --- |
| Template Matching | Rule-based anomaly detection | Simple, interpretable | Manual feature design, poor scalability | [9] |
| YOLOv5 | CNN-based object detection | Fast, reliable for large targets | Struggles with tiny lesions | [18,19] |
| DETR | Transformer-based end-to-end detection | Global context modeling | Poor small object detection | [22] |
| CDT-CAD | DETR with context-aware transformers | Effective for chest abnormalities | Complexity increases computation | [24] |

7. Cite the below publications in the paper:

>> https://doi.org/10.1016/j.imu.2021.100741
>> https://doi.org/10.1109/IICAIET55139.2022.9936837
>> https://doi.org/10.1109/ICCCNT56998.2023.10307223

**Response:** We thank for recommending these relevant publications. After reviewing them, we find they align with our work’s focus on deep learning for medical imaging and disease detection. We will integrate these citations into appropriate sections to enrich the literature context.

Recent advancements also include hybrid CNN approaches for pneumonia detection [18, 19] and deep learning models for lung disease classification [20], demonstrating the growing role of AI in thoracic diagnostics.

8. Improve the figure quality.

**Response:** We appreciate this feedback regarding the visual presentation of our work. The current figures may lack sufficient resolution or clarity in the provided document. We will enhance their quality by increasing resolution, improving font legibility, and ensuring consistent formatting.
